# Large multi-modal models – the present or future of artificial intelligence in medicine?

**DOI:** 10.3325/cmj.2024.65.1

**Published:** 2024-02

**Authors:** Zdenko Sonicki

On January 18, 2024, the World Health Organization (WHO) released the artificial intelligence (AI) ethics and governance guidance for large multi-modal models (LMM) (1,2). This new document, based on the WHO`s guidance published in June 2021, puts forward over 40 recommendations for appropriate use of LMMs, aiming to ensure protection and promotion of population health. However, it remains unclear how many people browsing the WHO website are completely familiar with LMM. We are in the early days of multimodal systems, so early that the abbreviation LMM is ambiguous and not completely accepted in scientific jargon. In statistical terminology, multimodal data means multimodal distribution, eg, bimodal distribution, and here it refers to the data type such as text, images, or audio. The WHO team has tried to clarify this by stating that AI refers to “the capability of algorithms integrated into systems and tools to learn from data so that they can perform automated tasks without explicit programming of every step by a human.” (1).

They seem to be suggesting, in line with the opinions of some AI scientists, that machine learning (ML) on big data are a synonym for AI. However, other researchers suggest that ML is just a part of AI, and that there is much more than ML within the AI scope. ML is the recognition of patterns and extraction of systematic information from data by computers, while AI is modeling of human intelligence by computer algorithms or using computers to perform tasks requiring human intelligence. Although the two definitions appear similar, they are not. When tasks performed by computers believed to require human intelligence become reliable and well-understood, it is not AI anymore, it is automation. In addition to ML, there are other parts of AI - automated reasoning (logical rule-based systems), knowledge engineering, robotics, and so on.

The WHO team explains the term generative AI as a category of AI techniques in which algorithms are trained on data sets that can be used to generate new content, such as text, images, or videos. In other words, LMMs are a form of generative AI that can generate diverse outputs not limited to the type of input fed into the algorithm. LMMs are predicted to be widely used in health care and scientific research.

The WHO`s media team points out that LMMs have been adopted faster than any consumer application in history. Since its launch in late 2022, ChatGPT has made a huge impact in AI promotion, and continues to impress and puzzle scientists and anyone else interested in contemporary technology, but is it really LMM? Chatbots like ChatGPT or Gemini are large language models. If additional modalities are added to large language models, they become LMMs. For example, if image inputs are added to a large language model, such as in GPT-4V, it becomes an LMM.

Importantly, not all multimodal systems are LMMs. A system can be called multimodal if its inputs are multimodal, if its outputs are multimodal, or if both its inputs and outputs are multimodal. However, to be designated as an LMM it needs to include a language model component.

Despite the confusing terminology, the WHO`s AI ethics and governance guidance discusses the potential benefits and risks of LMMs in health and medical care. Five broad applications of LMMs for health are outlined as follows: diagnosis and clinical care, patient-guided use, clerical and administrative tasks, medical and nursing education, and scientific research and drug development. The report also cautions against the risks of possible inaccurate or biased information generated by AI and highlights the harm that may be caused if such information is used when making health decisions. It also warns of automation bias, ie, a tendency of people to overlook errors made by AI or delegate difficult choices to LMM.

Finally, the document emphasizes the need for the engagement of governments, technology companies, health care providers, patients, and civil society in the deployment, oversight, and regulation of LMMs. Key recommendations are included for governments, which should be responsible for regulating LMMs. Governments should invest in not-for-profit or public infrastructure, use laws, policies, and regulations to ensure that LMMs meet ethical requirements and human rights standards; appoint a regulatory agency to approve LMMs; mandate post-release auditing assessments, etc. The guidance also includes recommendations for LMM developers to transparently design LMMs for all potential users, to perform well-defined tasks accurately and reliably, and to predict and understand potential secondary outcomes.

The AI ethics and governance guidance for LMMs is a very interesting document, useful and ahead of its time, available online for everyone interested in health care future and emerging problems. Perhaps, its strongest message is the following: “LMMs are unique in their mimicry of human communication and ability to carry out tasks they were not explicitly programmed to perform.” Mimicry is a huge step in evolutionary biology, referring to circumstances in which one species mimics or resembles another species in order to survive. In other words, it presents itself as someone or something different than it is. This is the reason why LMMs need scrutiny by all parties involved in health and medical care, and should be treated in the same way as a new drug or a new medical technology.

An unavoidable question is if AI is going to replace physicians and other health care professionals. LMMs are an AI tool, and tools extend our abilities. AI is a cognitive tool, and it extends our abilities in terms of what we can do, not to imitate what we do. This brings us to augmented intelligence in the future.

Intelligence is a human “mental quality that consists of the abilities to learn from experience, adapt to new situations, understand and handle abstract concepts, and use knowledge to manipulate one`s environment.” (3). We are changing our environment, influencing our extelligence. Maybe soon, we will be interested in and discuss artificial extelligence.
